# Effects of intimate partner violence and homophobic bullying on ART adherence among young Thai men who have sex with men: a causal mediation analysis

**DOI:** 10.21203/rs.3.rs-3704223/v1

**Published:** 2023-12-16

**Authors:** Doug H. Cheung, Alexis N. Reeves, Worawalan Waratworawan, Yamol Kongjareon, Thomas E. Guadamuz

**Affiliations:** Mahidol University; Stanford University; Mahidol University; Mahidol University; Mahidol University

**Keywords:** HIV/AIDS, Men who have sex with men, ART adherence, Victimization, Intimate partner violence, Thailand, Bullying, Depression, Longitudinal study, Causal mediation analysis

## Abstract

**Background:**

Adherence to antiretroviral therapy is crucial in determining health outcomes and secondary HIV transmission for people living with HIV/AIDS. Young men who have sex with men (YMSM) living with HIV are often challenged by the prevailing experiences of psychosocial stressors, such as intimate partner violence and homophobic bullying, which may negatively affect their HIV care engagement.

**Methods:**

This study is the first to utilize a prospective cohort design (N= 185) involving YMSM living with HIV in Thailand. We examined the effects of intimate partner violence and homophobic bullying on ART adherence. We also tested the mediating effect of depression on the relationship between intimate partner violence and homophobic bullying on ART adherence.

**Results:**

We found that intimate partner violence (AOR: 2.58, 95% CI: 1.13, 5.42) and homophobic bullying (AOR: 2.40, 95% CI: 1.26, 4.48) were associated with subsequent ART nonadherence. Moreover, depression partially mediated 17.4% (95% CI: 0.75%, 56%) of the effect of homophobic bullying on ART nonadherence.

**Conclusions:**

The results suggest that tailored interventions to optimize ART adherence should address the impacts of intimate partner violence and homophobic bullying for HIV+ YMSM. The screening and subsequent treatment of depression alone may not be sufficient to address the effects of intimate partner violence, homophobic bullying, and possibly other MSM-specific psychosocial stressors on ART adherence.

## Introduction

Adherence to antiretroviral therapy (ART) is pivotal to establishing and sustaining viral load suppression and preventing disease progression and secondary transmission among persons living with HIV. To meet the UNAIDS 95–95-95 targets to end AIDS, 95% of persons living with HIV should know their HIV status, 95% of those who know their HIV status should be on ART, and 95% of those on ART should be virally suppressed (1). Since 2014, ART has been available free of charge through national health insurance in Thailand (2). However, Thai men who have sex with men (MSM), a key population comprising 41% of all new infections, still have inadequate ART adherence and engagement in HIV care programs (3, 4). National data from Thailand found that only 79.5% of MSM who started ART remained in treatment a year later. Only 47% of Thai MSM self-rated their ART adherence as “very good” or “excellent” in the past 3 months (3, 4). Thus, significant public health efforts are needed to engage HIV + Thai MSM (1).

Young Thai MSM (YMSM; aged 18–24 years old) are disproportionately impacted by the HIV epidemic in Thailand (5, 6). With an elevated HIV incidence of 7.4 per 100 person-years between 2006 and 2014, YMSM is among the largest groups of newly HIV-infected subpopulations in Thailand (5). Young and adolescent MSM living with HIV present unique challenges to HIV care engagement (7, 8). More broadly, sexual and gender minorities experience heightened exposure to early life trauma and interpersonal violence, while YMSM could be especially vulnerable to these psychosocial stressors (9, 10). In particular, exposure to traumatic experiences and interpersonal violence may have unique impacts on YMSM living with HIV (11). Intimate partner violence has been found to be a barrier to ART adherence among women (12, 13). Hirshfield et al. found that MSM in the US with past-year IPV were more likely to have suboptimal engagement in HIV care and that those who reported past-year IPV were more likely to be younger (18–29 years old vs. 50+) (14). Furthermore, mental illness has been proposed as a potential mechanism by which IPV affects HIV-related health outcomes; however, it is unclear whether treatment of mental illness can improve IPV-related disruption of ART adherence (13).

Recent evidence suggests that experiences of bullying negatively affect ART adherence and other health-related outcomes among young PLWH in Sub-Saharan Africa (15–17). Homophobic bullying is another common form of trauma experienced by sexual and gender minorities. It is associated with a range of adverse health consequences, such as depression and posttraumatic stress disorder (PTSD) (18, 19). Despite being known as a “gay paradise” among international tourists and expats, youth and adolescents in Thailand endure pervasive homonegative social environments (20). A study in 2014 found that more than half of LGBT-identified secondary school Thai students have been bullied; bullied students were more likely to be depressed and suicidal (21, 22). YMSM living with HIV in Thailand may experience similar episodes of homophobic bullying, with negative mental health consequences that interrupt their ART adherence. However, research has yet to elucidate this relationship.

Worldwide evidence has shown that depression impedes ART adherence among PLWH (23). Evidence suggests that the detection and subsequent treatment of depression, whether through psychological intervention or the use of antidepressants, improves ART adherence (24, 25). MSM with HIV experience similarly elevated rates of depression; however, it is unclear whether treatment of depression would have a similar effect on ART adherence as seen in heterosexual persons living with HIV populations, considering the range of potentially unique psychosocial adversities experienced by MSM with HIV, e.g., IPV and homophobic bullying (26, 27). To elucidate the mechanisms underlying these psychosocial adversities and ART adherence, the proposed study sought to investigate the effects of IPV and homophobic bullying on ART adherence using a unique dataset consisting of a prospective cohort of YMSM living with HIV in Bangkok, which is also the first prospective cohort study ever conducted involving YMSM living with HIV from Thailand. In addition, we examined whether depression mediated the effects of IPV and homophobic bullying on ART adherence among study participants.

## Materials and methods

Data were drawn from a prospective cohort study of young MSM (aged 15–29 years old) living with HIV in Bangkok, Thailand. We recruited a total of 214 participants via the assistance of community-based organization (CBO) partners, including the Poz Home Center Foundation and Rainbow Sky Association of Thailand (RSAT). Eligibility criteria included (1) male sex at birth, (2) having had anal intercourse with a man in the past 6 months, (3) ability to speak Thai, (4) Thai nationality, (5) having lived in Bangkok for at least 6 months, (6) being between 15 and 29 years of age, (7) self-reporting an HIV-positive status, and (8) consenting to be followed up online every four months for a total period of 12 months. Between January and February 2018, we recruited eligible participants via a web-based survey using Qualtrics software (28). When participants completed the survey, they received 500 baht (17 USD) as compensation. Following the baseline survey, participants were asked to complete a follow-up survey at 4, 8, and 12 months. At the end of each survey administration, we asked participants whether they wanted to discuss personal concerns such as substance use and violence with a mental health professional. Upon receiving a positive response and subsequent consent, these participants were referred to appropriate mental health professionals. Informed consent was collected before participant enrollment. The Mahidol University Institutional Review Board reviewed and approved all study procedures (COA 2017/079.2803). This includes a waiver of parental permission for participants between the ages of 15 and 17. The Mahidol University Institutional Review Board granted this waiver on the grounds that more harm (e.g., unintended disclosure of sexual orientation or HIV status to parents or guardians) may occur to participants who are considered minors if parental permission is granted. The preceding evidence that supported our application for waiver of parental permission has been documented elsewhere (Field 29), as well as its applications to previously published (30–33). All participants between the ages of 15 and 17 were subjected to the same online informed consent procedures as those aged 18 or over. Data from this study are available by email with the corresponding author upon reasonable request.

### Measures

Participants self-reported the following sociodemographic characteristics: age (in years), employment status, educational level, average monthly income, sexual orientation, having a regular partner, and having received goods or opportunities (for example, money, drugs and alcohol, mobile phone, mobile phone credits, clothes, bags, grades or educational opportunities) in exchange for sex (i.e., engaged in sex work).

ART adherence was measured as the participant’s self-reported percentage of taking ART medications as prescribed over the past 7 days, from 0 to 100% with 1% increments. Prior studies have shown that an 80–90% adherence level is adequate for viral suppression (34). We categorized participants as ART nonadherent if they reported less than 90% adherence.

Depression was measured using the Center for Epidemiologic Studies Short Depression Scale (CESD-D-R), which assessed participants’ self-reported past 7 days of depression symptomology (35). The scale consists of 10 items, and a total depression score was calculated by summing all 10 items, with possible scores ranging from 0 to 30. A summed score equal to or above 10 indicates elevated depressive symptoms. The Cronbach alpha coefficient for this scale at baseline was 0.759, showing adequate internal consistency.

Intimate partner violence was assessed by asking the participants whether they had experienced the following experience in the past 6 months (past 4 months in subsequent follow-up surveys): being hurt, hit, slapped in the body by a regular partner, casual sex partner, or male sex work partner; being forced to have sex by a regular partner or casual sex partner; being forced to have sex during sex work being fondled; or having unwanted touching against their will. Participants reporting any of the above experiences were categorized as having experienced intimate partner violence.

Homophobic bullying was assessed by a single item asking whether the participants had ever been bullied (in the past 4 months in subsequent follow-up surveys) because of their sexual orientation or because they did not act like a boy/man.

### Data analysis

We conducted descriptive analyses using chi-square tests for categorical variables and two-sample t-tests for continuous variables to summarize and compare participant characteristics at baseline. First stratified by intimate partner violence in the past 6 months, then stratified by homophobic bullying. Since no missing values were reported among baseline and subsequent follow-up surveys, we used complete case analysis for all analyses.

Generalized linear mixed-effects models (GLMMs) were applied with the logit link function and unstructured covariance structure to estimate the prospective association between sociodemographic and psychosocial variables (depression, intimate partner violence, and homophobic bullying) and ART non-adherence. First, bivariate analyses were conducted between sociodemographic and psychosocial variables and ART nonadherence. Then, we estimated the adjusted parameters of the relationship between psychosocial variables (depression, intimate partner violence, and homophobic bullying) and ART nonadherence, adjusting for confounding effects within levels of age, education, income, regular partner status, and sex work. These confounding variables were identified a priori via literature reviews. Psychosocial variables, such as depression, IPV, and homophobic bullying, were not adjusted for in the multivariable models because they are conceptualized as mediators between each psychosocial variable and ART nonadherence (36).

We conducted causal mediation analyses on the mediating effects of depression on the relationship between intimate partner violence (or homophobic bullying) and ART nonadherence using the casual mediation analysis approach developed by Imai et al. (37). This approach utilizes a counterfactual framework assuming that (1) the exposure is independent of all potential values of the outcome and mediating variables within levels of confounding variables and (2) the observed mediator is independent of all potential outcomes within levels of confounding variables (37). The parameter estimates include the average direct effect (ADE), average causal mediation effect (ACME), total effect, and proportion mediated. The ADE describes the association between intimate partner violence (or homophobic bullying) and ART nonadherence in a scenario where levels of exposure to depression (the mediator) are similar among participants exposed to intimate partner violence (or homophobic bullying). The ACME describes the elevated odds of ART nonadherence among participants exposed to intimate partner violence (or homophobic bullying) as mediated by depression. The proportion mediated is calculated as the ratio of ACME to the total effect. This captures the importance of the mediator (i.e., % mediated via depression) in explaining the impact of the exposure on the outcome.

We conducted all analyses using R (version 4.3.1, R Foundation for Statistical Computing, Vienna, Australia). GLMMs were conducted using the “lme4” package in R (38)^,^ while causal mediation analyses were conducted using the “mediation” package in R (39).

## Results

Of the 214 eligible participants, 185 initiated ART at baseline and were included in the analysis. Three-fifths of the participants were aged 26 to 29 years old, 75.1% had a tertiary education or above, 55.7% were employed full-time, 34.1% made 15,001 baht or above, and most of the participants (95.1%) identified as gay. Nearly half (45.4%) of the participants reported having a regular partner, and a quarter (25.4%) reported ever providing sex work. Nearly half (43.2%) of participants reported a clinically significant level of depressive symptoms at baseline, 11.9% of participants reported being a victim of intimate partner violence at baseline, and 29.2% reported ever being subjected to homophobic bullying at baseline. Table 1 summarizes participant characteristics at baseline, stratified by status of intimate partner violence from the past 6 months and ever experienced homophobic bullying at baseline. Compared to participants who had not experienced intimate partner violence in the past 6 months at baseline, participants who experienced intimate partner violence in the past 6 months at baseline were more likely to have an education level of secondary or below (21.7% vs. 8.6%, p=0.02) and have symptoms of depression (17.5% vs 7.6%, p=0.04). Compared to participants who did not experience homophobic bullying, participants who experienced homophobic bullying were more likely to be depressed (46.4% vs 16.2%, p<0.001). Table 4 contains the bivariate analyses between sociodemographic variables and ART nonadherence. Participants with an income level between 5001 and 15,000 baht compared to those with 15,001 baht or above (OR: 2.26, 1.22, 4.43, p=0.01) were more likely to have ART nonadherence.

In our bivariate analyses (Table 2), we found that depression (OR: 2.07, 95% CI: 1.22, 3.59, p<0.001), intimate partner violence (OR: 2.41, 95% CI: 1.10, 4.88, p=0.02 and homophobic bullying (OR: 2.32, 95% CI: 1.23, 4.22, p<0.001) were significantly associated with ART nonadherence. After adjusting for age, education, income, regular partner status and sex work, depression (AOR: 2.29, 95% CI: 1.16, 4.65, p=0.01), intimate partner violence (AOR: 2.58, 95% CI: 1.13, 5.42, p=0.02), and homophobic bullying (AOR: 2.40, 95% CI: 1.26, 4.48, p=0.006) were significantly associated with ART nonadherence.

**Figure 1:**
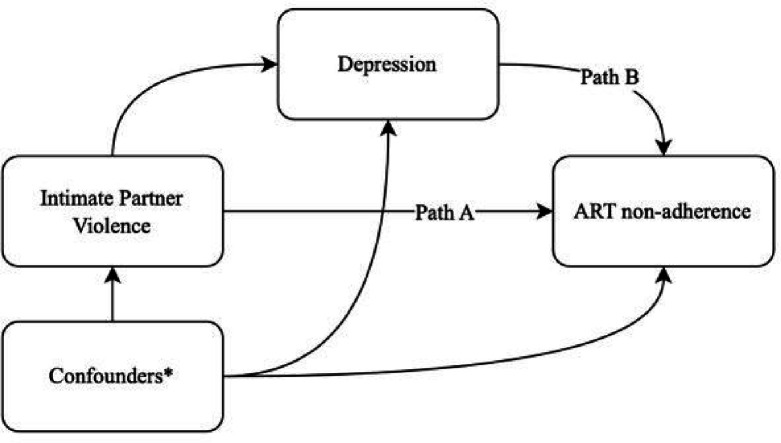
Directed acyclic graph (DAG) of the relationships between confounders, intimate partner violence, depression and ART nonadherence *Confounders include age, education, income, regular partner status and ever provided sex work

**Figure 2:**
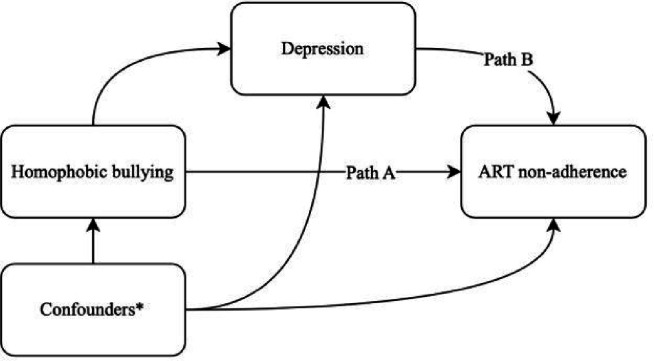
Directed acyclic graph (DAG) of the relationships between confounders, homophobic bullying, depression and ART nonadherence *Confounders include age, education, income, regular partner status and ever provided sex work

Table 3 presents parameter estimates from causal mediation analyses estimating the direct and indirect effects of intimate partner violence on ART nonadherence via depression and homophobic bullying on ART nonadherence via depression. In the first causal mediation analysis investigating the mediating role of depression on the relationship between intimate partner violence and ART nonadherence, intimate partner violence was positively associated with depression in the first step of the mediation model (AOR: 1.83, 95% CI: 1.11, 3.06, p=0.02). At the same time, depression was positively associated with ART nonadherence (AOR: 1.96, 95% CI: 1.13, 3.46, p=0.02). This resulted in an estimated average direct effect (ADE) of AOR:1.09 (95% CI: 1.01,1.22); however, we did not find evidence of an indirect effect since the average causal mediation effect (ACME) was not statistically significant (AOR: 1.01, 95% CI: 1.00, 1.02, p=0.09). In the second causal mediation analysis investigating the mediating role of depression on the relationship between homophobic bullying and ART nonadherence, homophobic bullying was positively associated with depression in the first step of the mediation model (AOR: 3.17, 95% CI: 2.09, 4.93, p<0.001). Meanwhile, depression was positively associated with ART nonadherence (AOR: 1.80, 95% CI: 1.02, 3.21, p=0.02). This resulted in an estimated ADE of AOR:1.07 (95% CI: 1.01, 1.15, p=0.03) and ACME of AOR:1.01 (95% CI: 1.00, 1.03, p= 0.03), suggesting that 17.4% (95% CI: 0.75%, 56%, p=0.04) of the effect of homophobic bullying on ART nonadherence was mediated by depression. The relationships between confounders (age, education, income, regular partner status, ever-provided sex work), exposures (intimate partner violence and homophobic bullying), mediators (depression), and outcome (ART nonadherence) were conceptualized by directed acyclic graphs (DAGs), as illustrated in Figure 1 and Figure 2, respectively.

In the sensitivity analyses to test for exposure mediator interaction, there was no significant interaction between IPV and depression on ART nonadherence or homophobic bullying and depression on ART nonadherence (Table 5).

## Discussion

In this prospective cohort study, we evaluated the longitudinal association between intimate partner violence, homophobic bullying, and ART nonadherence using a novel community study of young MSM with HIV living in Bangkok. Our results suggest that experiencing intimate partner violence and homophobic bullying are both associated with ART nonadherence. Moreover, we found that depression only partially mediated (17.4%, 95% CI: 0.75%, 56%) the relationship between homophobic bullying and ART adherence, while depression did not play a role in the relationship between IPV and ART adherence. Our results imply that a potential intervention for treating depression alone may not adequately address the effects of IPV and homophobic bullying on HIV-related health outcomes among young Thai MSM living with HIV. The results suggest that tailored interventions to improve HIV-related outcomes should address the multifaceted forms of victimization experienced by young HIV + MSM.

Among our cohort of young MSM with HIV living in Bangkok, those who experienced IPV were more likely to have suboptimal adherence to their ART regimen. This result is consistent with global evidence on the effect of IPV on ART adherence among HIV + women (12). Wang et al. found that among HIV + MSM in China, the verbal form of IPV was associated with a lower CD4 + cell count (40). In addition, the relationship between IPV and lower CD4 + cell count was fully mediated by depression. In contrast to this study, we found that depression did not play a significant mediating role in the relationship between IPV and ART adherence. This discrepancy could be because Wang et al.’s study examined the relationship with the verbal form of IPV, while our measures captured only physical and sexual forms of IPV. Depression may have differential mediating roles on different forms of IPV.

Additionally, depression may play a more vital role in determining the immunologic response (CD4 + cell count) than behaviors, e.g., ART adherence. A decrease in CD4 + cell count may account for an additional immunological impact of depression independent of ART adherence, which may explain the discrepancy in results across studies (41). Our result supports a direct relationship between IPV and ART adherence. MSM living with HIV who are experiencing IPV may have diminished self-efficacy to prioritize their health and self-care, as observed among heterosexual women with HIV (12).

Among our participants, those who experienced homophobic bullying were more likely to have suboptimal adherence to their ART regimen. Although the literature on this relationship is scant for HIV + MSM, our results are consistent with studies investigating the relationship between victimization and HIV-related outcomes among adolescents in sub-Saharan Africa (15, 42). Casale et al. found that psychological distress mediated the effect of bullying on ART adherence among adolescents living with HIV in South Africa (15). However, we found that only a relatively small fraction of the effect of homophobic bullying on ART adherence was mediated through depression. The effect of homophobic bullying on HIV-related outcomes can likely be mediated through other syndemic psychosocial factors, e.g., anxiety disorder, PTSD, and substance use (27). Harkness et al. found that childhood sexual abuse, PTSD, anxiety disorder, depression, alcohol abuse, and polysubstance/stimulant use each additively contributed to ART nonadherence among MSM (27). These syndemic psychosocial adversities often act in consortia in affecting MSM’s various health outcomes (27, 43, 44). To interrupt the negative health impacts of these syndemic sequelae, our study prompts the importance of developing and implementing tailored psychosocial interventions for HIV + MSM who have a history of being bullied, both to improve mental well-being and to optimize HIV-related outcomes.

In our prior publication, entailing the baseline data of this prospective cohort study, social support was negatively associated with ART nonadherence for those with increased depressive symptoms, suggesting social support’s protective role by interrupting the effect of depressive symptoms on ART nonadherence (45). Social support may also play a protective role in the relationships between IPV, homophobic bullying, and ART nonadherence. However, we cannot provide such analyses because social support was only measured at baseline. Future studies should investigate the protective roles of social support between a range of MSM-specific psychosocial stressors and exposures, such as internalized homophobia, experienced discrimination, HIV stigma, early life trauma, substance and alcohol use, etc., and HIV-related outcomes, including ART adherence.

The findings of this study should be interpreted in light of its limitations. First, we recruited participants through CBO referrals, which may not be representative of the underlying population of YMSM living with HIV in Thailand. Those engaged with CBOs were more likely to have fewer victimizing experiences, better ART adherence, and higher social and peer support, which may underestimate the relationship between our exposures and outcomes. Second, we used relatively simple measures for IPV and homophobic bullying, which may not adequately capture the full range of experiences of IPV and homophobic bullying. Such misclassification may underestimate the relationship between our exposures and outcome. Third, unobserved confounding might be present in the relationship between the mediator and outcome; for example, personality factors could be associated with depression and ART adherence (46). Such unmeasured confounding may violate the assumption of causal mediation analysis (37). Still, many critical confounding variables that may approximate some of these characteristics were measured and controlled for in this study. In our sensitivity analysis, after the inclusion of the exposure-mediator interaction on the relationship between homophobic bullying, depression, and ART nonadherence, the average causal mediation effect did not remain statistically significant. This is probably due to a lack of power from our limited sample size; however, since the exposure-mediator interaction was insignificant, we did not include it in our final analysis. Of note, only 2 participants were exposed to intimate partner violence and reported ART nonadherence at baseline. This sparsity in data may have led to a nonstructural violation of the positivity assumption, resulting in unstable or inaccurate estimates (47). Last, we did not adjust for possible observed and unobserved time-varying confounders. Potential time-varying confounders include prior depression level and prior ART adherence, which could bias associations in the positive direction. Future research should consider g-computation and instrumental variable approaches to account for time-varying confounding.

## Conclusion

Our study is novel in its contribution to the literature by using the first prospective cohort study involving YMSM living with HIV ever conducted in Thailand. We found that experiences of IPV and homophobic bullying impede ART adherence and that depression played a relatively small mediating role in the relationship between homophobic bullying and ART adherence. In Thailand, HIV care facilities are beginning to integrate mental health screening and treatment for persons living with HIV (48). Although the integration of mental health services into HIV care facilities is an essential first step, these programs can benefit from care providers who are competent to address MSM-specific psychosocial issues and their complex needs so that proper screening, treatment, and care plans can be devised to provide optimal HIV care for HIV + MSM (48, 49). Our results motivate tailored interventions addressing the impacts of victimizations on HIV + YMSM and highlight the importance of MSM-competent integrated HIV care to more holistically improve their HIV care continuum and mental health comorbidities.

## Figures and Tables

**Table 1.** Baseline characteristics of participants by intimate partner violence and homophobic bullying (N=185)

Characteristic	Overall (N = 185)		Exposure to Intimate partner violence in the past 6 months (n=22, 11.9%)			Exposure to Homophobic bullying (n=54, 29.2%)		
	n	(%)	n	(%)	*P* value	n	(%)	*P* value
Age								
15–25	37	(20.0)	5	(13.5)	0.733	8	(21.6)	0.26
26–29	148	(80.0)	17	(11.5)		46	(31.1)	
								
Education								
Secondary or below	46	(24.9)	10	(21.7)	0.02	10	(21.7)	0.20
Tertiary or above	139	(75.1)	12	(8.6)		44	(31.7)	
								
Employment								
Full-time	103	(55.7)	12	(117)	0.53	31	(30.1)	0.91
Part-time	38	(20.5)	3	(7.9)		10	(26.3)	
Unemployed or student	44	(23.8)	7	(15.9)		13	(29.5)	
								
Income								
5000 Bhat or below	35	(18.9)	4	(11.4)	0.76	21	(33.3)	0.54
5001–15000 Bhat	87	(47.0)	9	(10.3)		22	(25.3)	
15001 Bhat or above	63	(34.1)	9	(14.3)		11	(31.4)	
								
Sexual Orientation								
Gay	176	(95.1)	21	(11.9)	0.94	52	(29.5)	0.64
Bisexual	8	(4.9)	1	(11.1)		2	(22.2)	
Having a regular partner								
Yes	84	(45.4)	11	(13.1)	0.65	21	(25.0)	0.25
No	101	(54.6)	11	(10.9)		33	(32.7)	
								
Ever provided sex work								
Yes	47	(25.4)	8	(17.0)	0.21	18	(38.3)	0.11
No	138	(74.6)	14	(10.1)		36	(26.1)	
								
Depression								
Yes	80	(43.2)	14	(17.5)	0.04	37	**(46.4)**	**<0.001**
No	105	(56.8)	8	(7.6)		17	(16.2)	
								
ART nonadherence								
Yes	13	(7.0)	2	(15.4)	0.69	6	(46.2)	0.16
No	172	(93.0)	20	(11.6)		48	(27.9)	

Bolded = significant at p<0.05

**Table 2.** Associations between psychosocial variables and ART nonadherence.

	Unadjusted		Adjusted*	
	OR (95% CI)	*P*	AOR (95% CI)	*P*
Depression				
Yes	**2.07 (1.22–3.59)**	**<0.001**	**2.29 (1.16–4.65)**	**0.01**
No	Ref		Ref	
				
Intimate Partner Violence				
Yes	**2.41 (1.10–4.88)**	**0.02**	**2.58 (1.13–5.42)**	**0.02**
No	Ref		Ref	
				
Homophobic bullying				
Yes	**2.32 (1.23–4.22)**	**<0.001**	**2.40 (1.26–4.48)**	**0.006**
No	Ref		Ref	

* Models adjusted for age, education, income, regular partner status and ever provided sex work

Bolded = significant at p<0.05

**Table 3.** Causal mediation analyses of intimate partner violence and homophobic bullying on ART nonadherence via depression.

	AOR*	95% CI	*P* value
Intimate partner violence			
Intimate partner violence → Depression	**1.83**	**1.11, 3.06**	**0.02**
Depression → ART nonadherence	**1.96**	**1.13, 3.46**	**0.02**
Total effect	**1.10**	**1.02, 1.22**	**0.01**
Direct effect (not through depression, path A)	**1.09**	**1.01, 1.22**	**0.02**
Average causal mediation effect (through depression, path B)	1.01	0.99, 1.02	0.09
% Mediated	8.1 %	−1.5%, 39%	0.10
			
Homophobic bullying			
Homophobic bullying → Depression	**3.17**	**2.09, 4.93**	**<0.001**
Depression → ART nonadherence	**1.80**	**1.02, 3.21**	**0.02**
Total effect	**1.09**	**1.02, 1.17**	**0.008**
Direct effect (not through depression, path A)	**1.07**	**1.01, 1.15**	**0.03**
Average causal mediation effect (through depression, path B)	**1.01**	**1.00, 1.03**	**0.03**
% Mediated	**17.4%**	**0.75%, 56%**	**0.04**

* Models adjusted for age, education, income, regular partner status and ever provided sex work

Bolded = significant at p<0.05

**Table 4.** Bivariate associations of sociodemographic variable and ART nonadherence

	OR (95% CI)	*P* value
Age		
15–25	Ref	0.66
26–29	0.87 (0.48–1.68)	
		
Education		
Secondary or below	Ref	0.86
Tertiary or above	0.95 (0.52–1.82)	
		
Employment		
Full-time		
Part-time	1.38 (0.68–2.65)	0.35
Unemployed or student	1.34 (0.70–2.47)	0.36
		
Income		
5000 Bhat or below	1.43 (0.58–3.36)	0.42
5001–15000 Bhat	**2.26 (1.22–4.43)**	**0.01**
15001 Bhat or above	Ref	
		
Sexual Orientation		
Gay	2.31 (0.47–41.63)	0.42
Bisexual	Ref	
		
Having a regular partner		
Yes	1.37 (0.81–2.33)	0.24
No	Ref	
		
Ever provided sex work		
Yes	1.67 (0.84–3.14)	0.12
No		

Bolded = significant at p<0.05

**Table 5.** Sensitivity analyses assessing the impact of exposure-mediator interaction on casual mediation analyses’ parameter estimates.

	AOR*	95% CI	*P* value
Intimate partner violence			
Intimate partner violence → Depression	**1.83**	**1.11, 3.06**	**0.02**
Depression → ART nonadherence	**1.91**	**1.05, 3.54**	**0.04**
Depression * Intimate partner violence → ART nonadherence	1.20	0.25, 6.80	0.83
Total effect	**1.11**	**1.02, 1.22**	**0.01**
Direct effect (not through depression, path A)	**1.10**	**1.01, 1.22**	**0.03**
Average causal mediation effect (through depression, path B)	1.01	0.99, 1.03	0.23
% Mediated	**7.1%**	**−4%, 38%**	**0.24**
			
Homophobic bullying			
Homophobic bullying → Depression	**3.17**	**2.09, 4.93**	**<0.001**
Depression → ART nonadherence	1.82	0.96, 3.50	0.07
Depression * homophobic bullying → ART nonadherence	0.93	0.25, 4.07	0.92
Total effect	**1.08**	**1.02, 1.16**	**0.006**
Direct effect (not through depression, path A)	**1.07**	**1.01, 1.16**	**0.02**
Average causal mediation effect (through depression, path B)	1.01	0.99, 1.03	0.21
% Mediated	15.6%	−11.9%, 59%	0.21

* Models adjusted for age, education, income, regular partner status and ever provided sex work

Bolded = significant at p<0.05

## Abbreviations

YMSM: young men who have sex with men
UNAIDS: Joint United Nations Programme on HIV/AIDS
HIV: human immunodeficiency virus
PLWH: persons living with human immunodeficiency virus
CBO: community-based organization
ART: antiretroviral therapy
IPV: intimate partner violence
PTSD: posttraumatic stress disorder
CESD-D-R: Center for Epidemiologic Studies Short Depression Scale
ACME: average causal mediation effect
ADE: average direct effect
OR: odds ratio
AOR: adjusted odds ratio
CI: confidence interval
GLMMs: generalized linear mixed effects models
